# [^11^C]Metoclopramide PET can detect a seizure-induced up-regulation of cerebral P-glycoprotein in epilepsy patients

**DOI:** 10.1186/s12987-024-00588-8

**Published:** 2024-10-28

**Authors:** Myriam El Biali, Louise Breuil, Matthias Jackwerth, Severin Mairinger, Maria Weber, Michael Wölfl-Duchek, Karsten Bamminger, Ivo Rausch, Lukas Nics, Marcus Hacker, Sebastian Rodrigo, Viviane Bouilleret, Markus Zeitlinger, Ekaterina Pataraia, Nicolas Tournier, Martin Bauer, Oliver Langer

**Affiliations:** 1 Department of Clinical Pharmacology, Medical University of Vienna, Vienna, Austria; 2 Division of Clinical Pharmacology and Toxicology, Geneva University Hospitals, Geneva, Switzerland; 3 Laboratoire d’Imagerie Biomédicale Multimodale (BIOMAPS), Université Paris-Saclay, CEA, CNRS, Inserm, Service Hospitalier Frédéric Joliot, Orsay, France; 4 Department of Biomedical Imaging und Image-guided Therapy, Division of Nuclear Medicine, Medical University of Vienna, Vienna, Austria; 5 QIMP Team, Center for Medical Physics and Biomedical Engineering, Medical University of Vienna, Vienna, Austria; 6 Neurophysiologie et Epileptologie, Université Paris Saclay-APHP, Le Kremlin Bicêtre, Paris, France; 7 Department of Neurology, Medical University of Vienna, Vienna, Austria

**Keywords:** Blood-brain barrier, P-glycoprotein, Drug-resistant epilepsy, [^11^C]Metoclopramide, PET

## Abstract

**Background:**

P-glycoprotein (P-gp) is an efflux transporter which is abundantly expressed at the blood-brain barrier (BBB) and which has been implicated in the pathophysiology of various brain diseases. The radiolabelled antiemetic drug [^11^C]metoclopramide is a P-gp substrate for positron emission tomography (PET) imaging of P-gp function at the BBB. To assess whether [^11^C]metoclopramide can detect increased P-gp function in the human brain, we employed drug-resistant temporal lobe epilepsy (TLE) as a model disease with a well characterised, regional P-gp up-regulation at the BBB.

**Methods:**

Eight patients with drug-resistant (DRE) TLE, 5 seizure-free patients with drug-sensitive (DSE) focal epilepsy, and 15 healthy subjects underwent brain PET imaging with [^11^C]metoclopramide on a fully-integrated PET/MRI system. Concurrent with PET, arterial blood sampling was performed to generate a metabolite-corrected arterial plasma input function for kinetic modelling. The choroid plexus was outmasked on the PET images to remove signal contamination from the neighbouring hippocampus. Using a brain atlas, 10 temporal lobe sub-regions were defined and analysed with a 1-tissue-2-rate constant compartmental model to estimate the rate constants for radiotracer transfer from plasma to brain (*K*_1_) and from brain to plasma (*k*_2_), and the total volume of distribution (*V*_T_ = *K*_1_/*k*_2_).

**Results:**

DRE patients but not DSE patients showed significantly higher *k*_2_ values and a trend towards lower *V*_T_ values in several temporal lobe sub-regions located ipsilateral to the epileptic focus as compared to healthy subjects (*k*_2_: hippocampus: +34%, anterior temporal lobe, medial part: +28%, superior temporal gyrus, posterior part: +21%).

**Conclusions:**

[^11^C]Metoclopramide PET can detect a seizure-induced P-gp up-regulation in the epileptic brain. The efflux rate constant *k*_2_ seems to be the most sensitive parameter to measure increased P-gp function with [^11^C]metoclopramide. Our study provides evidence that disease-induced alterations in P-gp expression at the BBB can lead to changes in the distribution of a central nervous system-active drug to the human brain, which could affect the efficacy and/or safety of drugs. [^11^C]Metoclopramide PET may be used to assess or predict the contribution of increased P-gp function to drug resistance and disease pathophysiology in various brain diseases.

**Trial registration:**

EudraCT 2019-003137-42. Registered 28 February 2020.

**Supplementary Information:**

The online version contains supplementary material available at 10.1186/s12987-024-00588-8.

## Background

P-glycoprotein (P-gp, encoded in humans by the *ABCB1* gene and in rodents by the *Abcb1a* and *Abcb1b* genes) is an adenosine triphosphate-binding cassette (ABC) transporter which is abundantly expressed in the luminal (blood-facing) membrane of brain capillary endothelial cells forming the blood-brain barrier (BBB) [1]. P-gp accepts a broad range of endogenous and exogenous substances, including a variety of drugs, as its substrates. This transporter plays a crucial role in limiting the brain distribution of various drugs and can also clear endogenous substances, such as neurotoxic amyloid-beta peptides, from the brain into the blood [2, 3]. Changes in P-gp function at the BBB have been associated with several brain diseases, such as drug-resistant epilepsy, Alzheimer’s and Parkinson’s disease, brain tumours, stroke, multiple sclerosis, and amyotrophic lateral sclerosis [4].

Positron emission tomography (PET) with radiolabelled P-gp substrates has shown great promise to measure P-gp function at the human BBB [5]. The P-gp radiotracers which first became available (i.e., racemic [^11^C]verapamil, (*R*)-[^11^C]verapamil, and [^11^C]*N*-desmethyl-loperamide) were very efficiently transported by P-gp at the BBB, resulting in very low brain uptake and a limited sensitivity to detect moderate changes in P-gp function as they are expected to occur in various brain diseases [5]. This has led to the development of radiotracers which are “weak” P-gp substrates (i.e., [^11^C]metoclopramide and [^18^F]MC225) and which have higher brain uptake than previously described “avid” P-gp substrates [6, 7]. The radiolabelled antiemetic drug [^11^C]metoclopramide is transported by P-gp at the rat, mouse, non-human primate, and human BBB [6, 8–10]. Its human brain uptake is approximately three times higher than that of (*R*)-[^11^C]verapamil. We have shown that [^11^C]metoclopramide possesses a better sensitivity than (*R*)-[^11^C]verapamil and [^11^C]*N*-desmethyl-loperamide to detect a moderate (∼ 50%) reduction in P-gp expression at the BBB by employing heterozygous *Abcb1a/b* knockout mice (*Abcb1a/b*^*(+/−)*^) as an animal model [9]. We have also shown that [^11^C]metoclopramide can detect pharmacological P-gp induction in the mouse brain following treatment with the rodent pregnane X receptor activator 5-pregnen-3β-ol-20-one-16α-carbonitrile [11].

A large body of evidence shows that epileptic seizures lead to an up-regulation of P-gp at the BBB [12–14] *via* an intracellular signalling cascade triggered by release of the excitatory neurotransmitter glutamate [15]. Regional P-gp up-regulation in the epileptic brain forms the basis of the transporter hypothesis of drug resistance postulating restricted access of certain antiseizure medications (ASMs), which are transported by P-gp, to their pharmacological target sites in the brain [16, 17].

The aim of this study was to assess whether [^11^C]metoclopramide PET can detect increased P-gp function in the human brain. To this end, we employed drug-resistant temporal lobe epilepsy (TLE) as a model disease with a well-characterised, regional P-gp up-regulation in the temporal lobe (TL) [12–14] and compared the brain kinetics of [^11^C]metoclopramide between drug-resistant TLE patients (*n* = 8), drug-sensitive focal epilepsy patients (*n* = 5), and healthy subjects (*n* = 15).

## Methods

### General

This study was conducted in accordance with the Good Clinical Practice Guideline of the International Conference on Harmonisation and the Declaration of Helsinki. The trial was registered in the EudraCT database (2019-003137-42) and was approved by the Ethics Committee of the Medical University of Vienna and the Austrian Agency for Health and Food Safety. All subjects gave oral and written informed consent before enrolment in the study.

### Healthy subjects and patients

We included 15 healthy subjects (2 women, 13 men, age: 29 ± 11 years) and 13 epilepsy patients (5 women, 8 men, age: 33 ± 10 years) into our study. Healthy subjects were free of any medication for at least 14 days and judged as healthy based on clinical examination and routine blood and urine laboratory assessments. Patient characteristics are summarised in Supplementary Table 1. The diagnosis and focus localisation was based on prolonged video-electroencephalography (EEG) monitoring, a high-resolution 3T magnetic resonance imaging (MRI) scan using a predefined clinical epilepsy protocol, as well as [^18^F]FDG PET [18]. The patient group comprised 8 patients with drug-resistant focal TLE (DRE) and 5 patients with drug-sensitive focal epilepsy (DSE) who had been seizure-free for at least 1 year. The DRE patients fulfilled criteria of medical intractability [19]. Three of the DRE patients were MRI-negative (i.e., no epileptogenic lesion could be identified on MRI) and 5 had structural epilepsy, of whom 4 had hippocampal sclerosis (Supplementary Table 1). The majority (11/13) of the patients received at least one ASM for which cumulative literature evidence suggests transport by P-gp (e.g., phenytoin, phenobarbital, lamotrigine, oxcarbazepine, and levetiracetam) (Supplementary Table 1) [20].

### PET/MR imaging

Subjects underwent a PET/MRI brain scan on a fully-integrated PET/MRI system (Siemens Biograph mMR, Erlangen, Germany). A head and neck coil was used to ensure a high signal-to-noise ratio for the MR imaging. Foam cushions were placed inside the MR head coil to minimise involuntary head movement. The integrated PET/MR imaging protocol included a structural T1-weighted image acquired with a magnetisation prepared rapid gradient echo (MPRAGE) sequence (echo time/repetition time = 3.75/1.67 ms, inversion time = 950 ms, flip angle = 8°, 192 sagittal slices, voxel size = 1 × 1 × 1 mm) for spatial normalisation. Subjects were i.v. injected with [^11^C]metoclopramide (371 ± 36 MBq, containing < 20 μg of unlabelled metoclopramide, diluted to a final volume of 10 ml with physiological saline solution), which had been synthesised as described before [21], as a slow bolus over 20 s. At the start of the injection, a 60-min list mode PET data acquisition was initiated and arterial blood samples (3 ml) were manually collected approximately every 10 s for the first 2.5 min followed by 9-ml samples at 5, 10, 20, 30, 40, and 60 min after radiotracer injection. Aliquots of blood and plasma were measured for radioactivity in a gamma counter (Packard Cobra II auto-gamma counter, Packard Instrument Company, Meriden, Connecticut, USA), which was cross-calibrated with the PET/MRI scanner. The arterial plasma samples collected at 10, 20, 30, and 40 min after radiotracer injection were analysed for radiolabelled metabolites of [^11^C]metoclopramide with radio-high performance liquid chromatography as described elsewhere [10]. A mono-exponential decay function was fitted to the percentage of unchanged [^11^C]metoclopramide in plasma *versus* time and then applied to the decay-corrected total radioactivity counts in plasma to derive a metabolite-corrected arterial plasma input function. Following the PET/MRI examination, subjects were moved to a PET/CT system (Biograph Vision 600, Siemens Healthineers, Germany), where a low-dose computed tomography (CT) scan (120 kVp, 25 mAs) of the brain was acquired for the purpose of attenuation correction.

### Data analysis

**Fig. 1:**
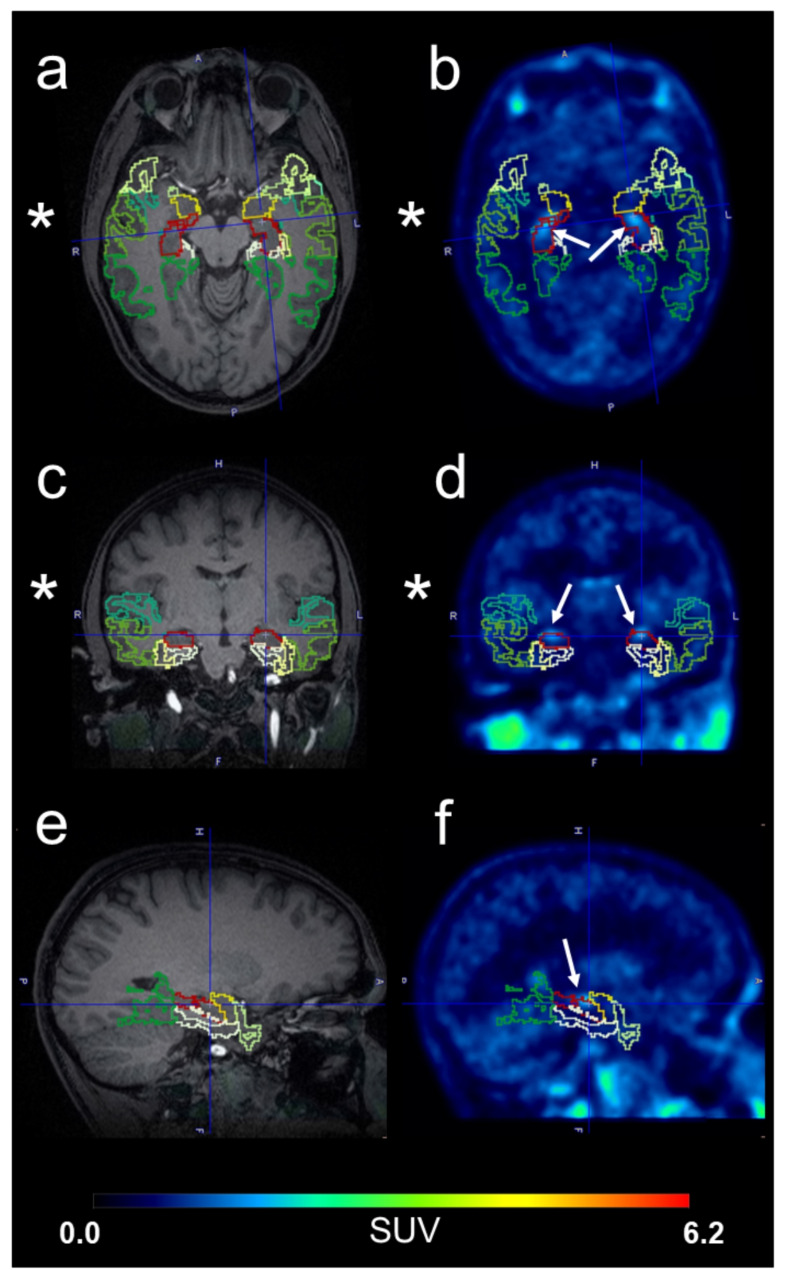
T1-weighted MR images (**a**,** c**,** e**) and averaged [^11^C]metoclopramide PET images (**b**,** d**,** f**) in 3 planes (from top to bottom: horizontal, coronal and sagittal [left hemisphere]) in one non-lesional drug-resistant epilepsy patient (p01-04) showing different temporal lobe sub-regions (green to yellow) from the brain region atlas (N30R83) and contamination of the hippocampus VOI (in red) by the choroid plexus (white arrows). The side of the seizure focus (right hemisphere) is indicated by an asterisk. Intensity scale for PET is expressed as standardised uptake value (SUV) and set from 0 to 6.2

The PET list mode data were re-binned into 1 × 15 s, 3 × 5 s, 3 × 10 s, 2 × 30 s, 3 × 60 s, 2 × 150 s, 2 × 300 s, and 4 × 600 s frames and each PET frame was reconstructed into a 256 × 256 × 127 matrix (voxel size 1.4 × 1.4 × 2.0 mm^3^) with an ordinary Poisson ordered subset expectation maximisation algorithm (OP-OSEM, 4 iterations, 21 subsets). A 3 mm Full Width at Half Maximum (FWHM) Gaussian post-reconstruction filter was applied to all images. Scatter correction along with a CT-attenuation correction was applied to all PET data. To perform the CT-attenuation correction, the low-dose CT scan was co-registered to the T1-MPRAGE sequence (RS-1) and a bilinear scaling was applied to convert the CT image to a CT-attenuation correction map. As the choroid plexus showed high radioactivity uptake resulting in spillover of radioactivity into the neighbouring hippocampus (Fig. 1), it was outmasked on PET average images [22, 23]. In brief, the choroid plexus was outlined on PET average images by interactive 3D iso-contouring using the PNEURO tool in PMOD (version 4.403, PMOD Technologies Ltd., Zürich, Switzerland). The “mask in” functionality was then used to replace the pixel values inside the outlined choroid plexus volume of interest (VOI) by the value zero on the dynamic PET images. The brain kinetics of [^11^C]metoclopramide were analysed using a brain region atlas (N30R83) [24] implemented in the PNEURO tool (Fig. 1). The anatomical MRI was segmented into grey and white matter and spatially normalised to a Montreal Neurological Institute T1-MRI template before transferring the atlas regions to the PET data. Time-activity curves (TACs) were extracted for the 10 TL sub-regions (left and right) included in the brain atlas, the entire temporal lobe as well as for whole brain grey matter. The atlas-derived hippocampal VOI was manually adjusted to remove the part contaminated by the choroid plexus signal.

The kinetic modelling tool PKIN implemented in PMOD was used to analyse the PET and metabolite-corrected plasma data employing a reversible 1-tissue-2-rate constant (1T2K) compartmental model to estimate the rate constants for radiotracer transfer from plasma to brain (*K*_1_, mL/(cm^3^ × min)), from brain to plasma (*k*_2_, 1/min), and the total volume of distribution (*V*_T_ = *K*_1_/*k*_2_, mL/cm^3^) [10]. The fractional arterial blood volume in the brain (*V*_b_) was included as a fitting parameter. For the regional modelling outcome parameters, asymmetry indices (AI) between the sides located ipsilateral and contralateral to the epileptic focus were calculated in patients using the formula 200% × [(ipsilateral – contralateral) / (ipsilateral + contralateral)] [22]. For healthy subjects, left-sided TL sub-regions were arbitrarily considered ipsilateral for calculation of AIs. For display purposes, the brain and metabolite-corrected plasma TACs were expressed in units of standardised uptake value (SUV). The area under the TACs (AUC, SUV × min) was calculated from 5 to 60 min after radiotracer injection using Prism (version 10.1.2, Graphpad Software, Dotmatics, Boston, MA, USA).

### Statistical analysis

The sample size of our study was based on feasibility and no sample size calculation was performed. Statistical analysis was performed using Prism. After confirmation of the normal distribution of the data using the Shapiro-Wilk normality test, outcome parameters were compared between two groups using two-sided, paired or unpaired t-tests and between multiple groups using one-way Analysis of Variance (ANOVA) followed by a Tukey’s multiple comparison test. For statistical comparison of modelling outcome parameters in different TL sub-regions ipsilateral and contralateral to the epileptic focus, the corresponding left-sided TL sub-regions of healthy subjects were used as control regions. The level of statistical significance was set to a *p* value of ≤ 0.05. All values are expressed as mean ± standard deviation (SD).

## Results

### Plasma analysis

**Fig. 2:**
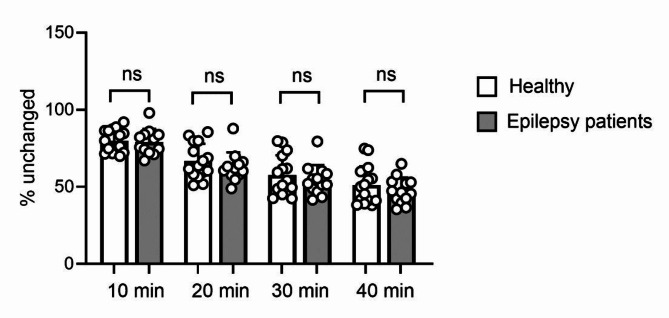
Percentage of unchanged [^11^C]metoclopramide in arterial plasma of healthy subjects (*n* = 15) and epilepsy patients (*n* = 13) at different time points after radiotracer injection. Error bars and lines indicate mean + SD. ns, not significant; two-sided, unpaired t-test

Total radioactivity counts in arterial plasma were corrected for radiolabelled metabolites of [^11^C]metoclopramide to generate arterial plasma input functions for kinetic modelling. In Supplementary Fig. 1, representative HPLC chromatograms of plasma samples obtained at 20 min after [^11^C]metoclopramide injection are shown for one healthy subject, one DRE and one DSE patient. The percentage of unchanged [^11^C]metoclopramide in arterial plasma was not significantly different between patients and healthy subjects at all examined time points after radiotracer injection (Fig. 2).

**Fig. 3:**
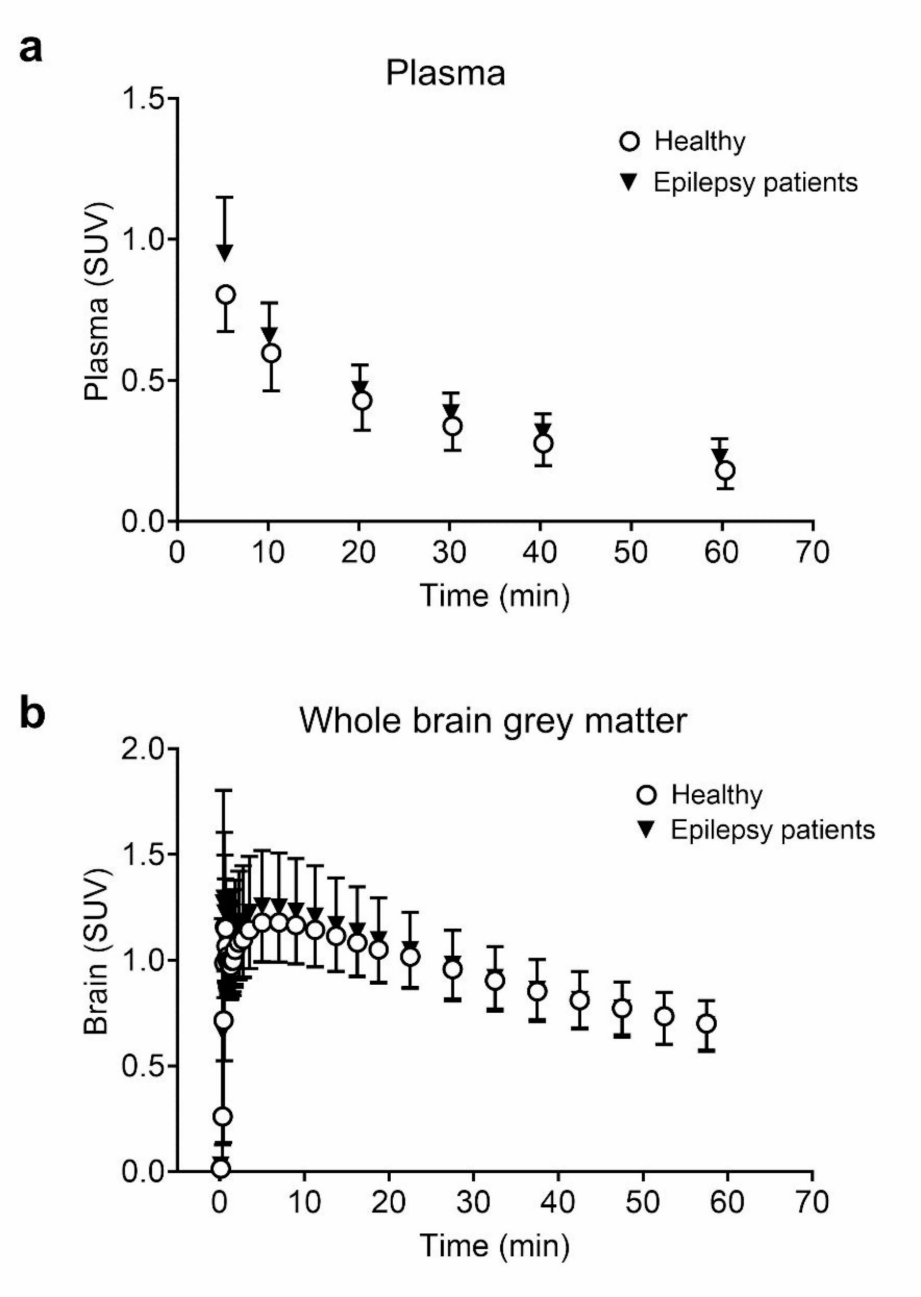
Time-activity curves (mean ± SD) of unchanged [^11^C]metoclopramide in arterial plasma (**a**) and total radioactivity in whole brain grey matter (**b**) of healthy subjects (*n* = 15) and epilepsy patients (*n* = 13)

TACs of unchanged [^11^C]metoclopramide in arterial plasma of patients and healthy subjects are shown in Fig. 3a. Despite comparable metabolism, the AUC of unchanged [^11^C]metoclopramide in arterial plasma was significantly higher in patients than in healthy subjects (AUC, patients: 22.8 ± 4.1 SUV × min, healthy: 19.4 ± 3.5 SUV × min, *p* ≤ 0.05).

### Brain analysis

TACs in whole brain grey matter of patients and healthy subjects are shown in Fig. 3b. Whole brain exposure to radioactivity was not significantly different between patients and healthy subjects (AUC, patients: 49.8 ± 8.9 SUV × min, healthy: 48.9 ± 7.4 SUV × min).

We used a brain region atlas to define 10 TL sub-regions and the entire TL ipsilateral and contralateral to the epileptic focus (Fig. 1). Since the high radioactivity signal in the choroid plexus contaminated the neighbouring hippocampus, the part contaminated by the choroid plexus signal was removed from the hippocampal VOI. The volumes of the hippocampal VOI before and after removal of the contaminated part are shown in Supplementary Fig. 2.

### Kinetic modelling

**Fig. 4:**
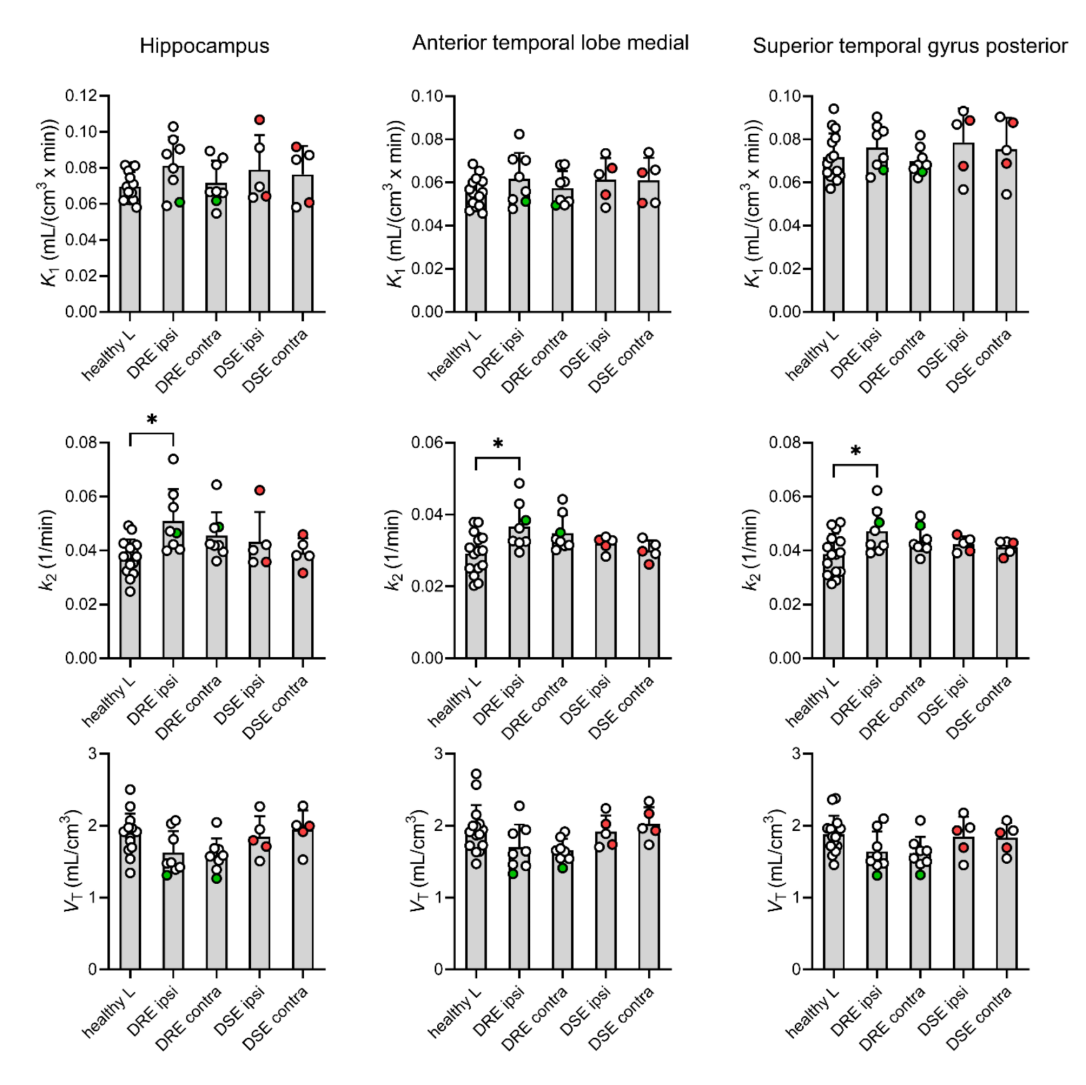
Outcome parameters from kinetic modelling (*K*_1_, *k*_2_ and *V*_T_) in the hippocampus, anterior temporal lobe, medial part and superior temporal gyrus, posterior part ipsilateral (ipsi) and contralateral (contra) to the epileptic focus of drug-resistant (DRE, *n* = 8) and drug-sensitive (DSE, *n* = 5) epilepsy patients as compared to corresponding left-sided control regions of healthy subjects (healthy L, *n* = 15). In two DSE patients (p03-02 and p03-03), the localisation of the epileptic focus was not known and was arbitrarily assigned to the left side (red symbols). In one DRE patient (p01-04), the epileptic focus was located temporal posterior (green symbols). Error bars and lines indicate mean + SD. *, *p* ≤ 0.05; one-way ANOVA followed by a Tukey’s multiple comparison test

PET data were analysed with a 1T2K model. A 2-tissue-4-rate constant compartmental model was also tried out to model the PET data, but this model failed to provide precise estimates of the *k*_4_ parameter in several subjects (data not shown). Regional modelling outcome parameters from the sides ipsilateral and contralateral to the epileptic focus of DRE and DSE patients obtained with the 1T2K model were statistically compared with the corresponding left-sided control regions of healthy subjects (Fig. 4 and Supplementary Table 2).

In 3 out of 10 TL sub-regions, significant differences were detected in DRE patients on the side located ipsilateral to the epileptic focus (Fig. 4). In the ipsilateral hippocampus (+ 34%), the ipsilateral anterior TL, medial part (+ 28%), and the ipsilateral superior temporal gyrus, posterior part (+ 21%), *k*_2_ values were significantly higher in DRE patients than in the corresponding left-sided control regions of healthy subjects. Moreover, in the contralateral anterior TL, lateral part of DRE patients *k*_2_ values were significantly increased (+ 24%) (not shown in Fig. 4, see Supplementary Table 2). The corresponding *V*_T_ values showed a clear trend towards decreases in DRE patients relative to healthy subjects without reaching statistical significance (Fig. 4). Outcome parameters in DSE patients were not significantly different from healthy subjects. For the entire TL, none of the modelling outcome parameters was significantly different among groups. In Supplementary Table 2, mean modelling outcome parameters are given for all 10 TL sub-regions and the entire TL. In general, regional *k*_2_ values were always higher and regional *V*_T_ values were always lower in DRE patients as compared with the corresponding left-sided control regions of healthy subjects, while they were similar to healthy subjects in DSE patients. These effects appeared to be bilateral in DRE patients (i.e. both ipsilateral and contralateral to the epileptic focus) and spread out across the entire TL.

### Asymmetry indices

**Fig. 5:**
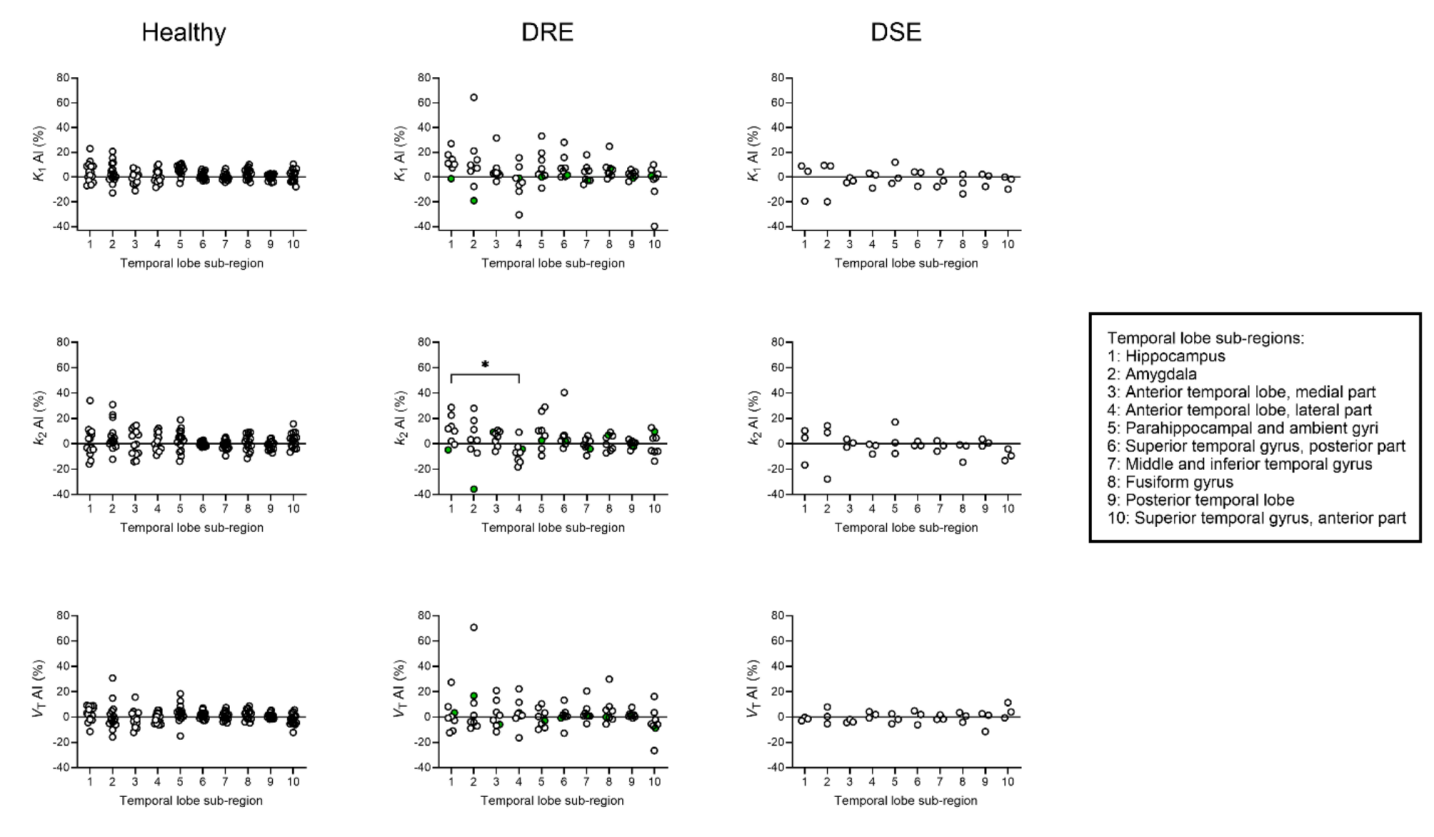
Asymmetry indices (AI, %) for outcome parameters from kinetic modelling (*K*_1_, *k*_2_ and *V*_T_) in 10 temporal lobe sub-regions of healthy subjects (*n* = 15), drug-resistant epilepsy (DRE) patients (*n* = 8) and drug-sensitive epilepsy (DSE) patients (*n* = 3). Two out of 5 DSE patients (p03-02 and p03-03) were excluded because the localisation of the epileptic focus was not known. In one DRE patient (p01-04), the epileptic focus was located temporal posterior (green symbols). *, *p* ≤ 0.05; one-way ANOVA followed by a Tukey’s multiple comparison test

We calculated asymmetry indices (AIs) between the sides ipsilateral and contralateral to the epileptic focus for the modelling outcome parameters from the 10 TL sub-regions (Fig. 5).

Regional AIs for all three outcome parameters appeared to be more pronounced in DRE patients than in healthy subjects and DSE patients (Fig. 5). Regional AIs of all three outcome parameters followed no consistent pattern in the DRE patients, that is they were positive in some patients and negative in others. However, in some TL sub-regions of DRE patients (e.g., hippocampus, amygdala, and parahippocampal and ambient gyri) AIs appeared to be more pronounced than in others. In the hippocampus of DRE patients, AIs of the *k*_2_ parameter were significantly different from those in the anterior TL, lateral part (Fig. 5).

## Discussion

In several brain diseases including epilepsy, up-regulation of cerebral P-gp occurs, which may decrease the brain distribution of drugs and thus contribute to drug resistance [4]. Immunohistochemical analysis of surgically resected brain tissue of drug-resistant TLE patients revealed an increased expression of P-gp in brain capillary endothelial cells of epileptogenic TL brain regions relative to normal brain tissue [12–14].

PET with radiolabelled P-gp substrates, such as (*R*)-[^11^C]verapamil, [^11^C]*N*-desmethyl-loperamide, [^18^F]MC225, and [^11^C]metoclopramide, has been used to measure P-gp function at the human BBB [10, 25–27]. The low brain uptake of (*R*)-[^11^C]verapamil and [^11^C]*N*-desmethyl-loperamide is not ideal for the detection of increased P-gp function, which will further decrease the imaging signal. We showed in a rat epilepsy model that the sensitivity of (*R*)-[^11^C]verapamil to detect P-gp up-regulation can be improved under conditions of partial P-gp inhibition with tariquidar [28]. This imaging protocol has been translated to humans by Feldmann et al., who performed paired (*R*)-[^11^C]verapamil PET scans in drug-resistant TLE patients and healthy subjects before and after partial P-gp inhibition [23]. This study showed for the first time an increase in P-gp function in different epileptogenic TL sub-regions of patients as reflected by an attenuated increase in (*R*)-[^11^C]verapamil *K*_1_, calculated from 0 to 10 min after radiotracer injection to minimise the influence of radiolabelled metabolites, in response to tariquidar. However, a routine clinical application of this imaging protocol is hampered by the lack of availability of tariquidar for clinical use and the safety risk associated with the potential interaction of tariquidar with concomitant medication. Moreover, (*R*)-[^11^C]verapamil is extensively metabolised by cytochrome P450 3 A (CYP3A) enzymes to radiolabelled metabolites that enter the brain [29]. Some ASMs (e.g., carbamazepine, phenytoin, and phenobarbital) can induce hepatic CYP3A enzymes leading to changes in the rate of (*R*)-[^11^C]verapamil metabolism [23, 30]. As altered radiotracer metabolism and brain entry of radiolabelled metabolites will impact the outcome parameters from kinetic modelling, this can confound the estimation of cerebral P-gp function with (*R*)-[^11^C]verapamil.

[^11^C]Metoclopramide has been designed as a radiotracer that overcomes the limitations of (*R*)-[^11^C]verapamil [6]. [^11^C]Metoclopramide has a higher passive permeability at the BBB than (*R*)-[^11^C]verapamil and therefore shows appreciable brain uptake despite P-gp-mediated efflux transport. In this aspect it resembles several P-gp-transported central nervous system-active drugs, such as phenytoin [31]. [^11^C]Metoclopramide is primarily metabolised by CYP2D6 [32], which is a non-inducible enzyme. This is supported by data in rats and healthy human subjects showing no effect of treatment with the potent CYP inducers carbamazepine or St. John´s wort on [^11^C]metoclopramide metabolism [33, 34]. In our study, [^11^C]metoclopramide metabolism was similar in epilepsy patients and healthy subjects (Fig. 2), but the majority of our patients was on non-enyzme-inducing ASMs (Supplementary Table 1). In the patient (p03-05) who was treated with carbamazepine, the AUC of unchanged [^11^C]metoclopramide in arterial plasma (21.6 SUV × min) was similar to the group mean (22.8 ± 4.1 SUV × min), which supported that [^11^C]metoclopramide metabolism is not inducible. Studies in rats showed that [^11^C]metoclopramide lacks brain-penetrant radiolabelled metabolites [6]. In humans, the major radiolabelled metabolite of [^11^C]metoclopramide in plasma has been identified as the corresponding *N*-*O*-glucuronide, which is expected, based on its chemical structure, to not penetrate the BBB [35]. However, a second, structurally unidentified radiolabelled metabolite was detected in human plasma (Supplementary Fig. 1), from which it is not known whether it can penetrate the human BBB.

To assess whether [^11^C]metoclopramide can detect P-gp up-regulation in the human epileptic brain, we compared the brain kinetics of [^11^C]metoclopramide in drug-resistant TLE patients, in whom the localisation of the epileptic focus was known, with healthy subjects and seizure-free, drug-sensitive epilepsy patients. We used a brain region atlas [24] to define different TL sub-regions located ipsilateral and contralateral to the epileptic focus (Fig. 1). Previously described P-gp substrate radiotracers ((*R*)-[^11^C]verapamil and [^11^C]*N*-desmethyl-loperamide) showed high radioactivity uptake in the choroid plexus, which resulted in spillover of radioactivity into the neighbouring hippocampus. The same phenomenon was observed for [^11^C]metoclopramide (Fig. 1). Similar to previous PET studies with (*R*)-[^11^C]verapamil [22, 23], we had to outmask the choroid plexus to remove the contaminated part from the hippocampal VOI (Supplementary Fig. 2). PET data were analysed with a 1T2K model. P-gp inhibition with cyclosporine A in humans led to an increase in *V*_T_ (= *K*_1_/*k*_2_) of [^11^C]metoclopramide (+ 29%), which was primarily caused by a decrease in *k*_2_ rather than an increase in *K*_1_ [10]. Conversely, P-gp induction is expected to result in an increase in *k*_2_ and a decrease in *V*_T_. *K*_1_ equals cerebral blood flow × extraction fraction and is therefore perfusion-dependent. It can be expected that a perfusion-dependent increase in *K*_1_ will lead to a concomitant increase in *k*_2_. We indeed observed a trend towards moderate bilateral *K*_1_ increases in the TL of both DRE and DSE patients (Supplementary Table 2). Since cerebral blood flow was not measured in our study, we have no proof that the observed moderate *K*_1_ increases were indeed due to increased cerebral blood flow. The *K*_1_ increases did not reach statistical significance in any investigated brain region and were in several epileptogenic TL sub-regions of DRE patients exceeded by *k*_2_ increases (Fig. 4). As *k*_2_ was increased to a larger extent than *K*_1_, regional *V*_T_ (= *K*_1_/*k*_2_) values were consistently decreased by 10–15% in all TL sub-regions of DRE patients as compared with healthy subjects (Supplementary Table 2). As opposed to the *k*_2_ increases the *V*_T_ decreases did not reach statistical significance, suggesting that *k*_2_ is a more sensitive parameter than *V*_T_ to measure increased P-gp function in the human brain with [^11^C]metoclopramide. Importantly, regional *k*_2_ increases and *V*_T_ decreases were not observed in seizure-free DSE patients, which supports the notion that P-gp up-regulation is a consequence of epileptic seizures.

Even though the *k*_2_ increases primarily reached statistical significance in sub-regions localised ipsilateral to the epileptic focus, they appeared to occur bilaterally, i.e. on both the ipsilateral and contralateral side (Fig. 4, Supplementary Table 2). This is further supported by AI values, which revealed no consistent asymmetry pattern between the ipsilateral and contralateral side (Fig. 5). While *k*_2_ AIs were predominantly positive (i.e. higher *k*_2_ values in the ipsilateral side) in some TL sub-regions (hippocampus, amygdala, and parahippocampal and ambient gyri), they were negative (e.g., anterior TL, lateral part) or close to zero in other regions. Similar observations have been made by Feldmann et al. in their (*R*)-[^11^C]verapamil PET study, in which (*R*)-[^11^C]verapamil *K*_1_ values were lower bilaterally in the TL of drug-resistant as compared to drug-sensitive TLE patients [23].

Our study provides evidence that disease-induced alterations in P-gp expression at the BBB can lead to changes in the distribution of a central nervous system-active drug to the human brain, which could affect the efficacy and/or safety of drugs. As such, our findings provide support for the transporter hypothesis of drug resistance in epilepsy [16, 17]. As P-gp-transported ASMs (e.g., phenytoin, phenobarbital, lamotrigine, oxcarbazepine, and levetiracetam) [20] can also be considered as “weak” P-gp substrates like metoclopramide, comparable reductions in their brain distribution may be expected in drug-resistant TLE patients as those observed for metoclopramide. The observed moderate reductions in the brain distribution of [^11^C]metoclopramide support the notion that P-gp up-regulation is not the only factor that contributes to drug resistance in epilepsy [16, 17]. An unexpected finding of our study is that P-gp function was bilaterally increased across several TL sub-regions beyond the immediate vicinity of the epileptic focus. This raises the possibility that the efficacy of certain P-gp-transported ASMs may be limited across the entire epileptic network and not just at the initial seizure focus. The diffuse P-gp up-regulation pattern in the TL limits the applicability of regional cerebral P-gp function as a diagnostic imaging biomarker to localise epileptic foci in the presurgical evaluation of DRE patients. However, [^11^C]metoclopramide PET may still provide clinically useful information by allowing to assess the contribution of P-gp up-regulation to drug resistance in different forms of epilepsy as well as in various other brain diseases (e.g. multiple sclerosis, brain tumours, and amyotrophic lateral sclerosis), which may entail the change of medication to non-P-gp transported drugs. A limitation of our [^11^C]metoclopramide PET protocol for a clinical application is the necessity to perform arterial blood sampling which is an invasive procedure. This calls for the future validation of simplified outcome parameters of cerebral P-gp function, that can be directly derived from the brain TACs without the need for arterial blood sampling [36].

## Conclusion

Our study showed that [^11^C]metoclopramide overcomes the limitations of previously employed P-gp substrate radiotracers and can detect a seizure-induced, regional P-gp up-regulation in the brains of drug-resistant TLE patients without the need to administer a P-gp inhibitor. The efflux rate constant *k*_2_ appeared to be the most sensitive outcome parameter from kinetic modelling to detect increased P-gp function with [^11^C]metoclopramide. [^11^C]Metoclopramide may find clinical application to assess or predict the contribution of increased P-gp function to drug resistance and disease pathophysiology in various brain diseases.

## Electronic supplementary material

Below is the link to the electronic supplementary material.

Supplementary Material 1

## Data Availability

The datasets generated during and/or analysed during the current study are available from the corresponding author on reasonable request (OL).

## Abbreviations

ABC: Adenosine triphosphate-binding cassette
AI: Asymmetry index
ASM: Antiseizure medication
AUC: Area under the curve
BBB: Blood-brain barrier
CT: Computed tomography
CYP: Cytochrome P450
DRE: Drug-resistant epilepsy
DSE: Drug-sensitive epilepsy
EEG: Electroencephalography
*K*_1_: Influx rate constant from plasma into brain
*k*_2_: Efflux rate constant from brain into plasma
MRI: Magnetic resonance imaging
PET: Positron emission tomography
P-gp: P-glycoprotein
SUV: Standardised uptake value
TAC: Time-activity curve
1T2K: 1-Tissue-2-rate constant
TL: Temporal lobe
TLE: Temporal lobe epilepsy
*V*_T_: Volume of distribution
VOI: Volume of interest
