# Supplementary material for: [11C]Metoclopramide PET can detect a seizure-induced up-regulation of cerebral P-glycoprotein in epilepsy patients
Source: Fluids Barriers CNS. 2024 Oct 28;21:87. doi: 10.1186/s12987-024-00588-8 (PMC11514750; doi:10.1186/s12987-024-00588-8)
Supplement: Supplementary file 1 — Supplementary Material 1 [file 12987_2024_588_MOESM1_ESM.docx]

**Supplementary Material 1**

**[^11^C]Metoclopramide PET can detect a seizure-induced up-regulation of cerebral P-glycoprotein in epilepsy patients**

Myriam El Biali, Louise Breuil, Matthias Jackwerth, Severin Mairinger, Maria Weber, Michael Wölfl-Duchek, Karsten Bamminger, Ivo Rausch, Lukas Nics, Marcus Hacker, Sebastian Rodrigo, Viviane Bouilleret, Markus Zeitlinger, Ekaterina Pataraia, Nicolas Tournier, Martin Bauer, Oliver Langer

**Supplementary Table 1** Clinical data of drug-resistant and drug-sensitive epilepsy patients.

| Patient | Group^a^ | Age,  years/sex | Duration of epilepsy (years) | Interval  last seizure to PET (days/years)^b^ | Average seizure frequency per month^c^ | Current ASMs  (dose: mg/day) | Focus localisation  (EEG) | MRI | Previous epilepsy surgery |
| --- | --- | --- | --- | --- | --- | --- | --- | --- | --- |
| p01-02 | 1 | 28/M | 9 | 42 | 3 | LAC (600), BRI (200), | R MT | No epileptogenic lesion | No |
| p01-04 | 1 | 25/M | unknown | 8 | 1 | LEV (3000), LAC (300) | R PT | No epileptogenic lesion | No |
| p02-01 | 2 | 24/M | 21 | 13 | 0^d^ | LEV (3000), LTG (200) | L T | HS L>R | No |
| p02-02 | 2 | 43/M | 13 | 21 | 30 | LAC (400), LEV (1000) | L MT | L TB encephalocele | No |
| p02-03 | 2 | 27/F | 8 | 10 | 2 | LAC (400), LEV (1500), CLB (5) | L T | HS L | Yes, ganglioma WHO grade I STG L |
| p02-05 | 2 | 47/M | 45 | 5 | 2 | LEV (2000), LAC (300) | R MT | HS R | No |
| p02-06 | 2 | 23/F | 18 | 9 | 10 | LEV (2000), LTG (400) | R HS | HS R | No |
| p04-03 | 4 | 43/M | 21 | 1 | 2 | OXC (1800), ZON (300), CEN (200) | BT MT | No epileptogenic lesion | Yes, SAH R |
| p03-02 | 3 | 25/M | 4 | 3 | 0 | LAC (200), LEV (2000) | BT | No epileptogenic lesion | No |
| p03-04 | 3 | 28/F | 10 | 3 | 0 | LAC (300), LEV (1750) | L MT | No epileptogenic lesion | No |
| p03-01 | 3 | 52/M | 38 | 3 | 0 | PER (4), LEV (3500), LTG (200), CLB (10) | L TPO | TPO L hemiatrophy and leukomalacia | No |
| p03-03 | 3 | 35/F | 3 | 1 | 0 | LEV (3000), LAC (300) | unknown | No epileptogenic lesion | No |
| p03-05 | 3 | 35/M | 11 | 1 | 0 | CBZ (600), LAC (300) | R F | No epileptogenic lesion | No |

^a^ Group 1: drug-resistant non-lesional focal epilepsy; group 2: drug-resistant epilepsy with epileptogenic structural lesion; group 3: focal epilepsy (lesional or non-lesional), seizure-free for at least 1 year; group 4: drug-resistant focal epilepsy (lesional or non-lesional) with multi-regional seizure onset zones

^b^ Interval given in days for drug-resistant patients and in years for seizure-free patients

^c^ Calculated over 12 months before the PET scan

^d^ 1-3 seizures per year

M = male; F = female; ASM = antiseizure medication; BRI = brivaracetam; CBZ = carbamazepine; CEN = cenobamat; CLB = clobazam; LAC = lacosamide; LTG = lamotrigin; LEV = levetiracetam; PER = perampanel; OXC = oxcarbazepine; ZON = zonisamid;

R = right; L = left; F = frontal; HS = hippocampal sclerosis; MT = mesio-temporal; TPO = temporo-parieto-occiptal; PT = posterior temporal; TB = temporo-basal; T = temporal; BT = bitemporal; STG = superior temporal gyrus; SAH = selective amygdalo-hippocampectomy

**Supplementary Table 2** Outcome parameters from kinetic modelling for different temporal lobe sub-regions and the entire temporal lobe (ipsilateral and contralateral to the epileptic focus) of drug-resistant (*n* = 8) and drug-sensitive (*n* = 5) epilepsy patients as compared to corresponding left-sided control regions of healthy subjects (*n* = 15).

| **Temporal lobe**  **sub-region** | **Group** | ***K*_1_ (mL/(cm^3^×min))** | ***k*_2_ (1/min)** | ***V*_T_ (mL/cm^3^)** | ***V*_b_** |
| --- | --- | --- | --- | --- | --- |
| Hippocampus | Healthy left | 0.070±0.008 (3-7) | 0.038±0.007 (6-16) | 1.89±0.28 (5-12) | 0.035±0.007 (5-15) |
|  | DRE ipsi | 0.081±0.016 (2-5) | 0.051±0.012 (5-9)* | 1.63±0.30 (2-6) | 0.042±0.010 (5-9) |
|  | DRE contra | 0.072±0.012 (3-5) | 0.045±0.009 (6-9) | 1.59±0.23 (1-7) | 0.044±0.009 (4-9) |
|  | DSE ipsi^a^ | 0.079±0.019 (3-4) | 0.043±0.011 (7-9) | 1.85±0.28 (2-6) | 0.041±0.007 (5-7) |
|  | DSE contra^a^ | 0.076±0.016 (3-4) | 0.039±0.005 (6-9) | 1.95±0.27 (4-7) | 0.040±0.007 (4-7) |
| Amygdala | Healthy left | 0.057±0.007 (4-9) | 0.030±0.007 (8-22) | 2.01±0.45 (6-17) | 0.035±0.008 (4-13) |
|  | DRE ipsi | 0.063±0.014 (2-8) | 0.036±0.010 (5-15) | 1.81±0.41 (4-11) | 0.044±0.012 (4-8) |
|  | DRE contra | 0.056±0.011 (4-7) | 0.035±0.008 (9-16) | 1.64±0.34 (6-12) | 0.046±0.018 (3-8) |
|  | DSE ipsi^a^ | 0.065±0.010 (3-5) | 0.034±0.003 (6-12) | 1.92±0.30 (4-9) | 0.037±0.005 (5-10) |
|  | DSE contra^a^ | 0.063±0.012 (4-6) | 0.032±0.005 (9-13) | 1.97±0.27 (7-10) | 0.038±0.004 (7-8) |
| Anterior temporal  lobe, medial part | Healthy left | 0.056±0.007 (2-5) | 0.029±0.006 (3-14) | 1.95±0.33 (2-11) | 0.036±0.008 (2-11) |
|  | DRE ipsi | 0.062±0.012 (2-4) | 0.037±0.006 (3-8)* | 1.70±0.32 (2-6) | 0.039±0.010 (3-6) |
|  | DRE contra | 0.057±0.008 (2-4) | 0.035±0.005 (4-8) | 1.66±0.16 (3-6) | 0.038±0.015 (3-6) |
|  | DSE ipsi^a^ | 0.061±0.010 (2-3) | 0.032±0.002 (3-7) | 1.92±0.22 (3-5) | 0.030±0.004 (3-6) |
|  | DSE contra^a^ | 0.061±0.010 (2-3) | 0.030±0.003 (4-6) | 2.03±0.23 (3-4) | 0.037±0.010 (3-4) |
| Anterior temporal  lobe, lateral part | Healthy left | 0.062±0.007 (2-5) | 0.033±0.005 (4-12) | 1.91±0.28 (2-7) | 0.024±0.007 (5-26) |
|  | DRE ipsi | 0.064±0.014 (2-4) | 0.038±0.007 (5-8) | 1.68±0.35 (3-6) | 0.025±0.007 (5-11) |
|  | DRE contra | 0.066±0.009 (3-4) | 0.041±0.007 (6-8)* | 1.62±0.23 (4-6) | 0.027±0.007 (6-9) |
|  | DSE ipsi^a^ | 0.071±0.013 (2-3) | 0.037±0.005 (3-6) | 1.91±0.28 (2-4) | 0.023±0.002 (5-7) |
|  | DSE contra^a^ | 0.069±0.009 (2-4) | 0.037±0.002 (5-9) | 1.87±0.25 (3-7) | 0.025±0.006 (6-9) |
| Parahippocampal and  ambient gyri | Healthy left | 0.061±0.008 (2-7) | 0.034±0.006 (4-14) | 1.83±0.25 (3-11) | 0.039±0.007 (3-12) |
|  | DRE ipsi | 0.064±0.012 (2-5) | 0.041±0.009 (4-10) | 1.60±0.28 (3-7) | 0.045±0.011 (3-5) |
|  | DRE contra | 0.059±0.010 (2-5) | 0.037±0.007 (5-11) | 1.60±0.22 (4-8) | 0.045±0.009 (3-6) |
|  | DSE ipsi^a^ | 0.066±0.012 (2-4) | 0.036±0.002 (5-9) | 1.80±0.26 (3-6) | 0.041±0.008 (3-5) |
|  | DSE contra^a^ | 0.063±0.010 (2-3) | 0.034±0.004 (5-7) | 1.88±0.29 (4-5) | 0.044±0.006 (2-5) |
| Superior temporal  gyrus, posterior part | Healthy left | 0.072±0.011 (2-6) | 0.039±0.007 (4-13) | 1.88±0.26 (2-9) | 0.039±0.009 (3-16) |
|  | DRE ipsi | 0.076±0.011 (1-3) | 0.047±0.008 (3-5)* | 1.64±0.28 (2-4) | 0.044±0.010 (2-4) |
|  | DRE contra | 0.070±0.006 (1-3) | 0.044±0.005 (3-6) | 1.62±0.23 (2-4) | 0.041±0.010 (2-4) |
|  | DSE ipsi^a^ | 0.079±0.016 (2-3) | 0.042±0.003 (3-6) | 1.85±0.28 (2-5) | 0.037±0.005 (3-5) |
|  | DSE contra^a^ | 0.076±0.013 (1-3) | 0.041±0.003 (3-6) | 1.83±0.21 (2-4) | 0.041±0.011 (2-4) |
| Middle and inferior  temporal gyrus | Healthy left | 0.067±0.009 (1-5) | 0.035±0.007 (3-12) | 1.98±0.32 (2-9) | 0.026±0.006 (3-13) |
|  | DRE ipsi | 0.071±0.012 (1-2) | 0.042±0.008 (2-5) | 1.74±0.31 (1-3) | 0.029±0.006 (3-5) |
|  | DRE contra | 0.069±0.008 (1-2) | 0.042±0.007 (2-4) | 1.67±0.22 (2-3) | 0.029±0.006 (3-5) |
|  | DSE ipsi^a^ | 0.075±0.015 (1-2) | 0.038±0.003 (2-5) | 1.96±0.27 (2-3) | 0.027±0.004 (3-5) |
|  | DSE contra^a^ | 0.073±0.011 (1-2) | 0.037±0.003 (2-4) | 1.96±0.24 (2-3) | 0.026±0.002 (3-4) |
| Fusiform gyrus | Healthy left | 0.062±0.008 (2-7) | 0.032±0.006 (5-17) | 1.96±0.29 (2-12) | 0.026±0.005 (4-16) |
|  | DRE ipsi | 0.066±0.009 (2-4) | 0.038±0.006 (4-8) | 1.76±0.33 (3-6) | 0.031±0.008 (4-7) |
|  | DRE contra | 0.062±0.008 (2-3) | 0.037±0.005 (4-7) | 1.66±0.18 (3-5) | 0.029±0.002 (4-7) |
|  | DSE ipsi^a^ | 0.066±0.013 (2-4) | 0.034±0.002 (4-9) | 1.97±0.33 (3-7) | 0.032±0.005 (4-6) |
|  | DSE contra^a^ | 0.065±0.012 (2-5) | 0.033±0.004 (4-11) | 1.99±0.26 (3-8) | 0.034±0.009 (3-12) |
| Posterior temporal  lobe | Healthy left | 0.070±0.010 (1-5) | 0.038±0.007 (2-11) | 1.89±0.27 (2-8) | 0.030±0.007 (2-18) |
|  | DRE ipsi | 0.074±0.009 (1-2) | 0.045±0.007 (1-4) | 1.67±0.26 (1-3) | 0.035±0.007 (2-3) |
|  | DRE contra | 0.072±0.008 (1-2) | 0.045±0.007 (2-4) | 1.64±0.25 (1-3) | 0.036±0.005 (2-4) |
|  | DSE ipsi^a^ | 0.077±0.016 (1-2) | 0.041±0.002 (2.7-3.4) | 1.86±0.32 (2-2.5) | 0.032±0.006 (2.7-3.3) |
|  | DSE contra^a^ | 0.076±0.013 (1-2) | 0.041±0.003 (2-3) | 1.85±0.24 (1-2) | 0.030±0.001 (2-3) |
| Superior temporal  gyrus, anterior part | Healthy left | 0.060±0.008 (2-7) | 0.034±0.006 (5-16) | 1.81±0.23 (3-12) | 0.040±0.010 (3-14) |
|  | DRE ipsi | 0.059±0.012 (3-8) | 0.039±0.008 (7-17) | 1.54±0.40 (4-12) | 0.048±0.015 (4-6) |
|  | DRE contra | 0.061±0.008 (3-4) | 0.039±0.007 (6-8) | 1.59±0.25 (4-6) | 0.045±0.015 (4-7) |
|  | DSE ipsi^a^ | 0.065±0.012 (2-3) | 0.036±0.005 (3-7) | 1.81±0.24 (2-5) | 0.034±0.005 (3-5) |
|  | DSE contra^a^ | 0.064±0.009 (2-3) | 0.035±0.003 (5-7) | 1.82±0.16 (4-5) | 0.038±0.008 (3-6) |
| Entire temporal  lobe | Healthy left | 0.067±0.009 (1-6) | 0.036±0.007 (2-12) | 1.90±0.27 (2-9) | 0.032±0.006 (2-16) |
|  | DRE ipsi | 0.071±0.010 (1-2) | 0.043±0.008 (2-4) | 1.68±0.28 (1-3) | 0.036±0.007 (1-3) |
|  | DRE contra | 0.068±0.008 (1-2) | 0.042±0.007 (2-4) | 1.63±0.23 (1-3) | 0.036±0.006 (2-3) |
|  | DSE ipsi^a^ | 0.074±0.014 (1-1.5) | 0.039±0.003 (2-3) | 1.87±0.28 (1-2) | 0.032±0.005 (2-3) |
|  | DSE contra^a^ | 0.072±0.012 (0.9-1.3) | 0.038±0.003 (2-3) | 1.89±0.23 (1-2) | 0.033±0.003 (2-3) |

Values are reported as mean ± standard deviation. The values in parentheses represent the precision of the parameter estimates (expressed as range of standard error in percent). DRE, drug-resistant epilepsy; DSE, drug-sensitive epilepsy; ipsi, ipsilateral to the epileptic focus; contra, contralateral to the epileptic focus; *K*_1_ (mL/(cm^3^×min)), rate constant for radiotracer transfer from plasma to brain; *k*_2_ (1/min), rate constant for radiotracer transfer from brain to plasma; *V*_T_ (mL/cm^3^), total volume of distribution; *V*_b_, fractional arterial blood volume

^a^ In 2 out of 5 DSE patients (p03-02 and p03-03) the localisation of the epileptic focus was not known and was arbitrarily assigned to the left side

* *p* ≤ 0.05 for comparison with left-sided control region in healthy subjects using one-way ANOVA followed by a Tukey’s multiple comparison test


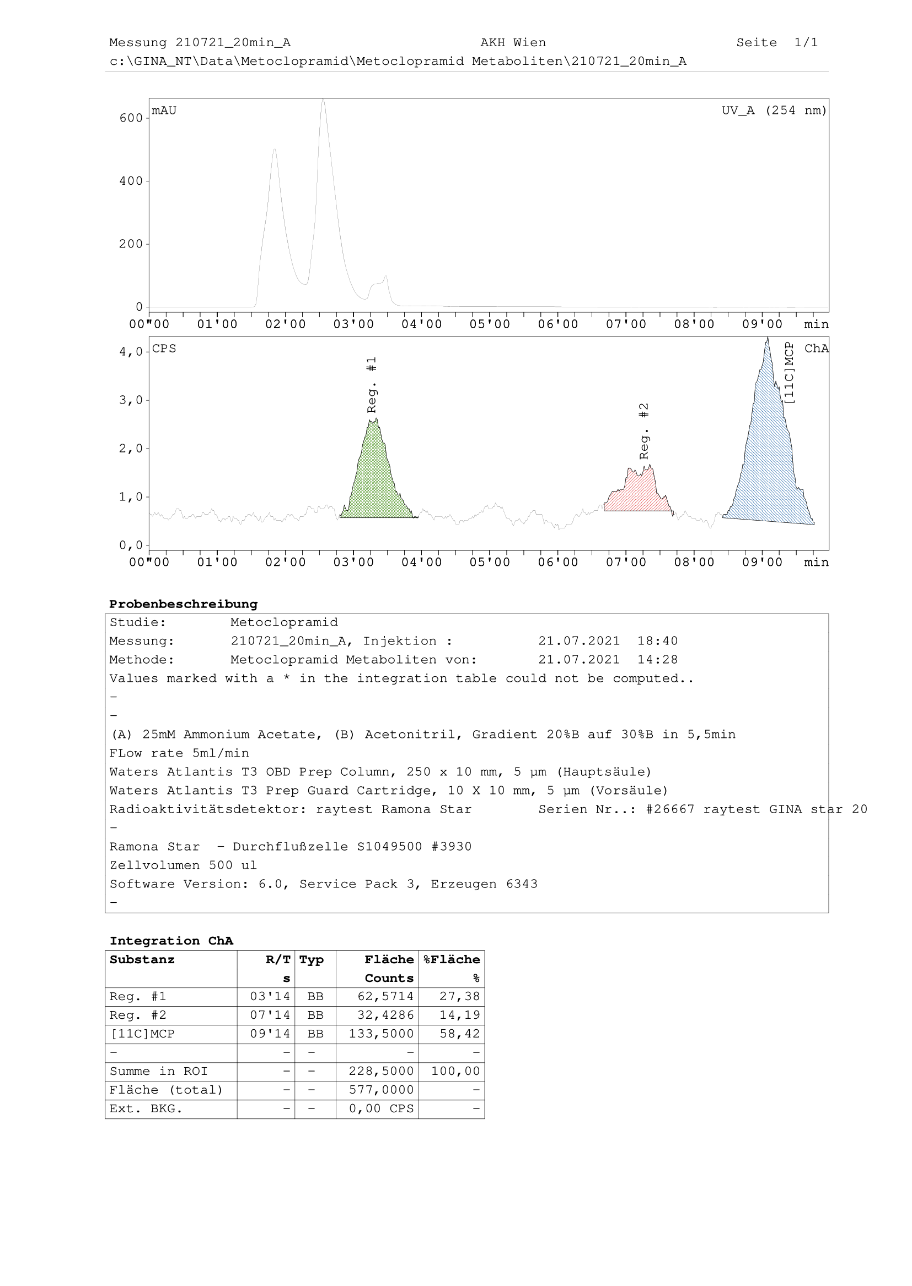
**a**


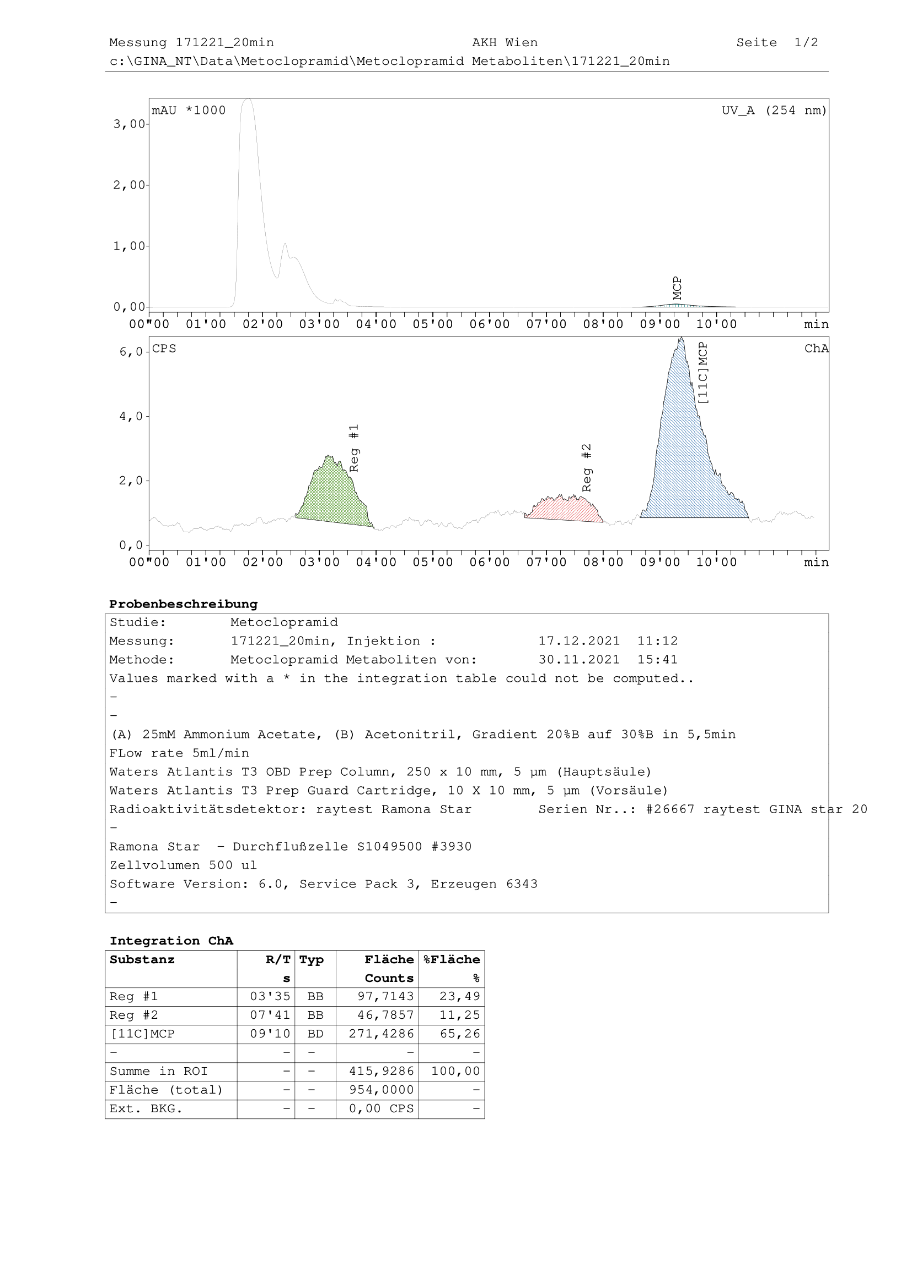
**b**

**
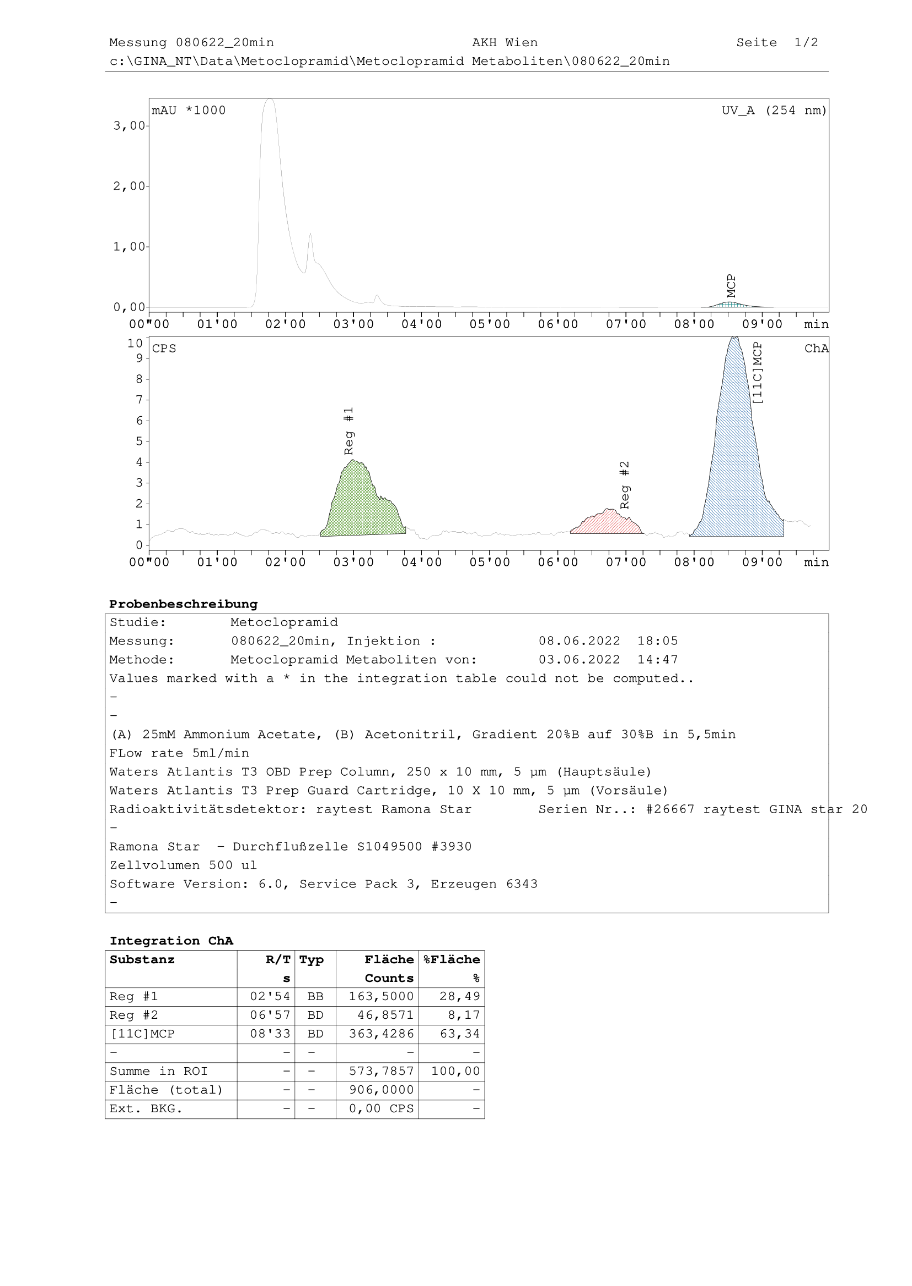
c**

**Supplementary Figure 1.** Representative radio-HPLC chromatograms for analysis of plasma obtained at 20 min after [^11^C]metoclopramide injection in one healthy subject (**a**), one drug-resistant (**b**) and one drug-sensitive epilepsy patient (**c**). The upper channel represents UV absorption (254 nm) and the lower channel radioactivity detection. Unchanged parent ([^11^C]MCP) elutes with a retention time of approximately 9 min. In addition, two radiolabelled metabolites eluting at approximately 3 min and 7 min were detected. The metabolite eluting at 3 min has been identified as the *N*-*O*-glucuronide of [^11^C]metoclopramide.





**Supplementary Figure 2.** Volume (cm^3^) of left and right hippocampus VOI before (HL and HR) and after (HL CORR and HR CORR) removal of the the part contaminated by the choroid plexus signal in healthy subjects (*n* = 15), drug-resistant epilepsy (DRE) patients (*n* = 8) and drug-sensitive epilepsy (DSE) patients (*n* = 5). Error bars and lines indicate mean + SD.
